# The human parasite *Loa loa *in cytokine and cytokine receptor gene knock out BALB/c mice: survival, development and localization

**DOI:** 10.1186/1756-3305-5-43

**Published:** 2012-02-21

**Authors:** Nicholas Tendongfor, Samuel Wanji, Julius C Ngwa, Mathias E Esum, Sabine Specht, Peter Enyong, Klaus I Matthaei, Achim Hoerauf

**Affiliations:** 1 Department of Microbiology and Parasitology, University of Buea, PO Box 63, Buea, Cameroon; 2 Research Foundation for Tropical Diseases and Environment, Buea, Cameroon. PO Box 474; 3 Institute of Medical Microbiology, Immunology and Parasitology (IMMIP), University Hospital Bonn, Sigmund-Freud Str. 25, 53105 Bonn, Germany; 4 Tropical Medicine Research Station, PO Box 55, Kumba, Cameroon; 5 Stem Cell & Gene Targeting Laboratory, The John Curtin School of Medical Research, The Australian National University Canberra, Australia

**Keywords:** BALB/c *mice*, knock out gene, cytokine, *L. loa*, survival, development, recovery rate, localization.

## Abstract

**Background:**

Immunological mechanisms involved in the survival and development of human filarial species in the vertebrate host are poorly known due to the lack of suitable experimental models. In order to understand the role of cytokines in the survival and development of filarial larvae in the vertebrate host, we infected different strains of BALB/c mice deficient in a number of cytokine or cytokine receptor genes with *Loa loa*. The survival and development of larvae were monitored.

**Methods:**

BALB/c mice genetically deficient in IL-4R, IFN-γ, IFN-γ/IL-5, IL-5, and IL-4R/IL-5 cytokine or cytokine receptor genes were infected with a human strain of *L. loa *and necropsies were performed at different time intervals up to 70 days post infection to monitor the survival and development of *L. loa *larvae. The larvae were teased out of the skin, muscles, peritoneal and pleural cavities, heart and lung tissues. The length and width of the recovered larvae were measured to assess their growth.

**Results:**

In mice deficient for IL-4R, IFN-γ, IFN-γ/IL-5, IL-5 and IL-4R/IL-5, the larvae survived up to 5, 20, 40, 50 and 70 days respectively. Worms recovered 70 days post infection in IL-4R/IL-5 DKO mice were young adults and measured 10.12 mm in length and 0.1 mm in width. Overall, 47% of larvae were recovered from subcutaneous tissues, 40% from muscles, 6% from the peritoneal cavity and 4% from the pleural cavity, lungs and heart.

**Conclusion:**

*L. loa *exhibits a differential survival and development in different strains of cytokine or cytokine receptor gene knockout mice with IL-4R and IL-5 playing critical roles in the host resistance to *L. loa *infection. The knock out BALB/c mouse therefore represents a useful tool to explore the key effectors of adaptive immunity involved in the killing of the *L. loa *parasite in a mammal host.

## Background

Loiasis is a neglected tropical disease that has recently emerged as a disease of public health importance due to its negative impact on the control of onchocerciasis in areas where the two infections co-exist. Individuals harboring a heavy microfilarial load of *L. loa *develop severe adverse events (SAEs) following ivermectin treatment [1]. Presently there is no satisfactory treatment for loiasis. Both ivermectin and diethylcarbamazine induce SAEs [2-4]. Pending the development of new chemotherapeutic molecules to treat loiasis, an alternative control method that has been less explored for loiasis is the development of a vaccine. Such a control tool, which could prevent the production of infection or inhibit the production of microfilariae by female worms, could be an ideal solution for the control of loiasis. But this will be possible only through a better understanding of the immune response induced by the parasite in the host and the identification of major effectors of such immune responses.

A major obstacle facing research on loiasis and particularly vaccine development has been the lack of suitable animal models. Apart from humans, *L. loa *develops up to patency in baboons and drills. These non human primate models have been used to study the biology of *L. loa *in the mammal host [5-7] and some components of the immune interactions between *L. loa *and the host [8-11]. However, due to ethical considerations and difficulties in handling these primates, they are not conducive for laboratory experimentation. Experimentation with laboratory mice is more practical since they can easily be handled and their genetic composition has been well characterized. Unfortunately, *L. loa *does not undergo a full course of development in laboratory wild-type mice. In filariasis, knockout mice have been used as tools for the study of the host immune response [12-15]. In experiments to infect rodents with *L. loa*, it was observed that infective larvae survived for only 1 week in immune-competent BALB/c and Swiss mice, whereas in the same mice immune-depressed with hydrocortisone, *L. loa *larvae survived for up to 3 weeks [16]. This indicates the role of the immune response in the clearance of *L. loa *from the murine host. In order to improve on the knowledge of the biology of *L. loa *in the mammalian host and to better understand the role of cytokines in the survival and development of *L. loa *in the mammalian host, we infected 5 strains of BALB/c mice deficient in specific cytokines (or a cytokine receptor) with human *L. loa *and the survival and development of the larvae were monitored.

## Methods

### Mice

The mice used in the experiment were obtained from the Institute of Medical Microbiology, Immunology and Parasitology (IMMIP), University of Bonn, Germany, where the following strains had been used and described before [17]:

(i) BALB/c IFN-γ^-/- ^mice, originally purchased from The Jackson Laboratory (Bar Harbor, Maine, USA); (ii) BALB/c IL-5^-/- ^mice [18], (iii) BALB/c IFN-γ ^-/-^/IL-5^-/- ^mice; (iv) BALB/c IL-4Rα^-/-^mice [19] and (v) BALB/c IL-4Rα^-/-^/IL-5^-/-^, which were provided by K.I.M [20] (henceforth known as IL-4R/IL-5 DKO).

All mice used for the experiments were reared and infected in the laboratory of the Research Foundation for Tropical Diseases and Environment, Buea, Cameroon.

### Ethical considerations

Mice used in the study were handled in accordance with the international guiding principles for biomedical research involving animals. This also involved a cross-check of the animal handling protocols provided by the Research Foundation for Tropical Diseases and Environment, for compliance with German regulations of animal husbandry by the chief veterinarian of the animal facilities at the Medical Facilities of Bonn University. An ethical clearance for the involvement of humans in the study as donors of microfilariae was obtained from the ethical committee of the Tropical Medicine Research Station Kumba and the ethical committee of the University of Bonn, Germany. Informed consent was obtained from all human participants.

### Parasites and infection of mice

*L. loa *of human origin was used in the study. Infective larvae used to infect the mice were obtained from *Chrysops silacea *engorged on a microfilaraemic volunteer. The engorged flies were kept in captivity for 12 - 14 days and fed on 15% sucrose (supplemented with 500 U penicillin and 500 μg/ml streptomycin), maintained at 23 - 28°C and 79% - 80% relative humidity. The flies were then dissected in RPMI 1640 medium (Sigma) and the infective larvae collected, counted and concentrated in 100 - 200 μl RPMI 1640. The mice were each inoculated with 100 L3s subcutaneously in the right lumbar area.

### Necropsy and larval recovery

Infected animals were dissected at different time intervals (2, 5, 10, 15, 20, 30, 40, 50, 60, 70 days post infection). The protocol used in the dissection of mice was described in previous studies [21,16]. Animals were dissected in RPMI medium and the larvae were teased out from the skin, muscles, peritoneal and pleural cavities, heart and lung tissues. The larvae recovered were counted and the site of recovery noted [22]. Recovered larvae were fixed in 5% formalin for measurement.

### Development and growth of *L. loa *in mice

The length and width of larvae recovered from different strains at different time points were measured using a microscope fitted with a Camera Lucida. L3s dissected from *Chrysops *were measured and served as a baseline to calculate larval growth.

### Data analysis

The mean number of larvae recovered at different necropsy time was generated and a two way analysis of variance (ANOVA) was used to compare the mean recovery at different time points in different strains. The proportions of larvae recovered from each organ were generated and compared using the Chi-square test. The Benferroni Post Hoc test was used to compare the increase in length and width of larvae recovered in different strains of mice at different time points after log transformation to normalize the data. All the statistical tests were performed at a 5% significance level.

## Results

### Survival of *L. loa *larvae in single and double cytokine/cytokine (receptor) knockout mice

**Figure 1:**
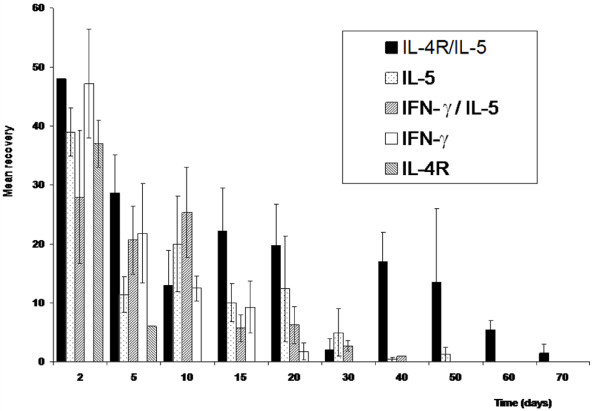
**Mean number of L. *loa *worms recovered at different necropsy times in single (IFN-γ ^-/-^, IL-5 ^-/- ^, 14 IL4R ^-/-^) and double knockout (IL-4/IL-5 ^-/- ^and INF-γ/IL-5 ^-/-^) BALB/c mice**. Each mouse was inoculated with 100 L3s of *L. loa *and necropsy was done at different time period. The bars represent the Standard error of the mean.

In all the five strains investigated, the number of larvae recovered dropped with time (Figure 1). A drastic drop in larval recovery was observed during the first 2 days of infection with less than 50% of larvae recovered. Overall, the mean number of larvae recovered differed significantly (p < 0.05) from one strain of mice to another. IL-4R^-/-^/IL-5^-/- ^DKO supported the infection better than any other strain (70 days), followed by IL-5^-/- ^KO (50 days), IFN-γ^-/-^/IL-5^-/- ^DKO (40 days), IFN-γ^-/- ^KO (20 days) and lastly IL-4R^-/- ^KO (5 days). Within each mouse strain, a significant difference (IL-5^-/- ^KO (P < 0.001), IFN-γ^-/- ^(P < 0.001), IFN-γ^-/-^/IL-5^-/- ^(P < 0.05) and IL-4R^-/-^/IL-5^-/- ^(P < 0.05)) was observed in the mean number of larvae recovered at different time points.

### Migration and localization of *L. loa *worms

In both single and double cytokine/cytokine receptor knockout mice, larvae were recovered from the subcutaneous tissues, muscles, peritoneal and pleural cavities, heart and lungs. More than 80% of larvae were recovered from the muscle and subcutaneous tissues in all strains of mice (Table 1). With the exception of IL-4R^-/-^/IL-5^-/- ^DKO mice, there was a significant difference (p < 0.05) between the proportion of larvae recovered from the subcutaneous tissue and the muscles. In IL-4R^-/-^/IL-5^-/- ^DKO and IL-4R^-/- ^KO mice, more larvae were recovered from the muscles, whereas in the other strains of mice more larvae were recovered from the subcutaneous tissue.

**Table 1:** Recovery rate and localization of larvae recovered from different organs of knockout BALB/C mice experimentally infected with human *Loa loa*.

Strain	No. of animal dissected	No. of larvae recovered	Subcutaneous tissue	Muscle	Genital organ	Peritoneal cavity	Pleural cavity	Heart and Lungs	Statistics
IL-4 R-α	14	80	23(28.75)	49(61.25)	5(6.25)	2(2.50)	1(1.25)	0	-

INF-γ KO	34	430	296(68.84)	73(16.98)	14(3.26)	25(5.81)	16(3.72)	6(1.40)	χ^2 ^= 1058.92 p = 0.0001

IL-5 KO	48	524	254(48.47)	207(39.50)	9(1.72)	34(6.49)	14(2.67)	6(1.15)	χ^2 ^= 866.30 p = 0.0001

INF-γ/IL-5 DKO	23	283	137(48.41)	112(39.58)	3(1.06)	23(8.13)	7(2.47)	1(0.35)	χ^2 ^= 472.02 p = 0.0001

IL-4/IL-5 D KO	27	439	174(39.64)	190(43.28)	5(1.14)	31(7.06)	29(6.61)	10(2.28)	χ^2 ^= 593.43 p = 0.0001

**Statistics**			χ^2 ^= 95.12 P = 0.0001	χ^2 ^= 104.13 P = 0.0001	χ^2 ^= 13.64 P = 0.0001	χ^2 ^= 3.87 P = 042	χ^2 ^= 14.04 P = 0.007	-	

Each mouse was inoculated with 100 L3 of *L. loa*. The figures in brackets are the percentages of larvae recovered.

Larvae migrated rapidly from the site of inoculation (subcutaneous tissue) to muscles, and internal organs. Larvae were found in the peritoneal and pleural cavities two days after inoculation. Larvae reached the heart and the lungs by day 5 in IFN-γ^-/- ^KO, day 10 in IFN-γ^-/-^/IL-5^-/- ^DKO and IL-5^-/- ^KO and day 15 in IL-4R^-/-^/IL-5^-/- ^DKO (Table 2). In IFN-γ^-/-^/IL-5^-/- ^DKO larvae recovered 40 days post infection were all from the subcutaneous tissues whereas in IFN-γ^-/- ^KO mice, larvae recovered 20 days post infection were from the subcutaneous tissues, muscles and genital organs. Larvae recovered 70 days post infections in IL-4R^-/-^/IL-5^-/- ^DKO were from the heart and muscles.

**Table 2:** Mean number of larvae recovered at different time point in different organs of BALB/c knockout mice experimentally infected with human *Loa loa*. The numbers in brackets are mean recovery.

Strain	DPI	D2	D5	D10	D15	D20	D30	D40	D50	D60	D70	Total
	**Nb of mice dissected**	**2**	**1**	**2**	**2**	**4**	**2**	**1**				**14**
	
	**Nb of larvae recovered**	74	6	0	0	0	0	0				80
	
	**Sub_Cut**	23 (11.5)	0	0	0	0	0	0				23(1.6)
	
	**Muscle**	43(21.5)	6 (6)	0	0	0	0	0				49(3.5)
	
**IL-4R KO**	**GO**	5(2.5)	0	0	0	0	0	0				5(0.3)
	
	**Peri-Cav**	2(1.0)	0	0	0	0	0	0				2(0.1)
	
	**Plu-Cav**	1(0.5)	0	0	0	0	0	0				1(0.1)
	
	**Heart/Lung**	0	0	0	0	0	0	0				0
	
	**Total**	74(6.2)	6(1.2)	0	0	0	0	0				

	**Nb of mice dissected**	**5**	**5**	**4**	**3**	**4**	**3**	**3**	**4**	**3**		**34**
	
	**Nb of larvae recovered**	236	109	50	28	7	0	0	0	0		430
	
	**Sub_Cut**	188(37.6)	64(12.8)	27(6.8)	14(4.7)	3(0.8)	0	0	0	0		296(8.7)
	
	**Muscle**	29(5.8)	32(6.4)	8(2.0)	2(0.7)	2(0.5)	0	0	0	0		73(2.1)
	
**IFN-γ KO**	**GO**	7(1.4)	2(0.4)	2(0.5)	1(0.3)	2(0.5)	0	0	0	0		14(0.4)
	
	**Peri-Cav**	10(2.0)	4(0.8)	5(1.3)	6(2.0)	0	0	0	0	0		25(0.7)
	
	**Plu-Cav**	2(0.4)	5(1.0)	5(1.3)	4(1.3)	0	0	0	0	0		16(0.5)
	
	**Heart/Lung**	0	2(0.4)	3(0.8)	1(0.3)	0	0	0	0	0		6(0.2)
	
	**Total**	236(7.9)	109(3.6)	50(2.1)	28(1.6)	7(0.3)	0	0	0	0		

	**Nb of mice dissected**	**3**	**3**	**3**	**4**	**4**	**4**	**2**	**2**			**25**
	
	**Nb of larvae recovered**	84	62	76	23	25	11	2				283
	
	**Sub_Cut**	58(19.3)	25(8.3)	31(10.3)	10(2.5)	8(2.0)	3(0.8)	2(1)	0			137(5.5)
	
	**Muscle**	22(7.3)	32(10.7)	29(9.7)	10(2.5)	11(2.8)	8(2.0)	0	0			112(4.5)
	
**IFN-γ/IL-5 DKO**	**GO**	0	0	2(0.7)	0	1(0.3)	0	0	0			3(0.1)
	
	**Peri-Cav**	4(1.3)	5(1.7)	7(2.3)	2(0.5)	5(1.3)	0	0	0			23(0.9)
	
	**Plu-Cav**	0	0	6(2.0)	1(0.3)	0	0	0	0			7(0.3)
	
	**Heart/Lung**	0	0	1(0.3)	0	0	0	0	0			1(0.0)
	
	**Total**	84(4.7)	62(3.5)	76(4.2)	23(0.9)	25(1.1)	11(0.5)	2(0.2)	0			

	**Nb of mice dissected**	**5**	**7**	**5**	**6**	**5**	**4**	**5**	**4**	**2**	**5**	**48**
	
	**Nb of larvae recovered**	195	80	100	60	62	20	2	5	0	0	524
	
	**Sub_Cut**	145(29.0)	36(5.1)	34(6.8)	19(3.2)	12(2.4)	7(1.8)	0	1(0.3)	0	0	254(4.9)
	
	**Muscle**	29(5.8)	38(5.4)	51(10.2)	31(5.2)	41(8.2)	13(3.3)	2(0.4)	2(0.5)	0	0	207(3.9)
	
**IL-5 KO**	**GO**	5(1.0)	2(0.3)	0.0	2(0.3)	0	0	0	0	0	0	9(0.2)
	
	**Peri-Cav**	14(2.8)	2(0.3)	4(0.8)	4(0.7)	9(1.8)	0	0	1(0.3)	0	0	34(0.7)
	
	**Plu-Cav**	2(0.4)	2(0.3)	8(1.6)	1(0.2)	0	0	0	1(0.3)	0	0	14(0.3)
	
	**Heart/Lung**	0	0	3(0.6)	3(0.5)	0	0	0	0	0	0	6(0.1)
	
	**Total**	195(6.5)	80(1.9)	100(3.3)	60(1.7)	62(1.8)	20(0.8)	2(0.1)	5(0.2)	0	0	524(1.6)

	**Nb of mice dissected**	**1**	**3**	**3**	**4**	**4**	**3**	**3**	**2**	**2**	**2**	**27**
	
	**Nb of larvae recovered**	48	86	39	89	79	6	51	27	11	3	439
	
	**Sub_Cut**	35(35)	53(17.7)	15(5.0)	36(9.0)	22(5.5)	1(0.3)	7(2.3)	1(0.5)	4(2.0)	0.0	174(7.7)
	
	**Muscle**	10(10)	23(7.7	23(7.6)	31(7.8)	34(8.5)	5(1.7)	36(12.0)	23(11.5)	3(1.5)	2(1.0)	190(6.9)
	
**IL-4/IL-5 DKO**	**GO**	0	2(0.7)	0	1(0.3)	1(0.3)	0	1(0.3)	0	0	0	5(0.1)
	
	**Peri-Cav**	2(2)	6(2.0)	1(0.3)	10(2.5)	7(1.8)	0	4(1.3)	1(0.5)	0	0	31(0.8)
	
	**Plu-Cav**	1(1)	2(0.7)	0	9(2.3)	12(3.0)	0	3(1.0)	1(0.5)	1(0.5)	0	29(0.9)
	
	**Heart/Lung**	0	0	0	2(0.5)	3(0.8)	0	0	1(0.5)	3(1.5)	1(0.5)	10(0.4)
	
	**Total**	48(8)	86(2.2)	39(2.2)	89(3.7)	79(3.3)	51(0.3)	51(2.7)	27(2.3)	11(0.9)	3(0.3)	439(2.6)

DPI: day post infection; Sub_Cut: Subcutaneous tissue; GO: Genital organ; Peri-Cav: peritoneal cavity; Plu-Cav: Pleural cavity. The figures in brackets are the mean larvae recovered which represent the total number of larvae recovered in each organ divided by the number of animals dissected.

### Growth and development of *L. loa *in different strains of BALB/c mice

**Figure 2:**
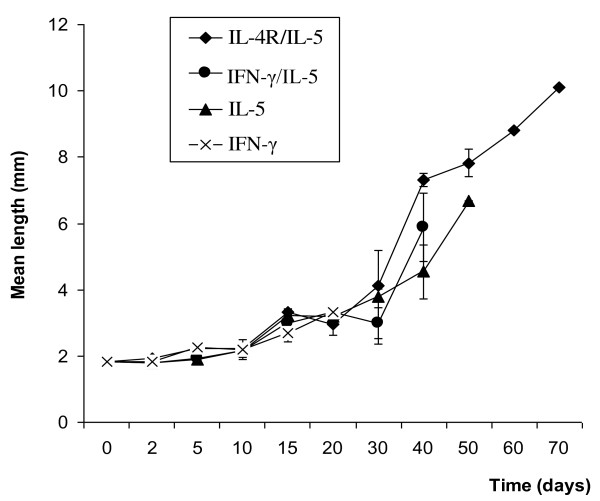
**Mean length (mm) of *L. loa *worms recovered in BALB/c mice deficient in cytokine/cytokine receptor genes after necropsy**. The bars represent the Standard error of the mean.

**Figure 3:**
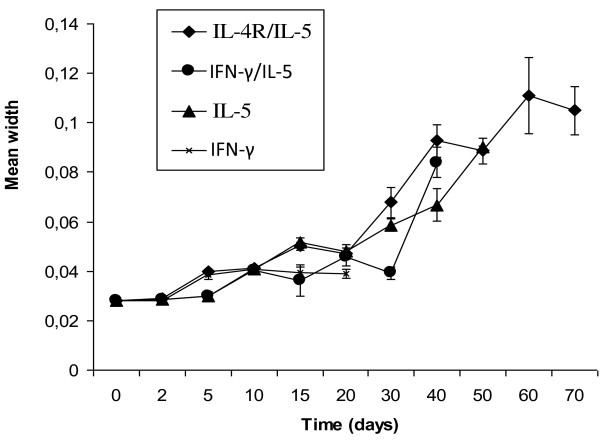
**Mean width (mm) of *L. loa *worms recovered in BALB/c mice deficient in cytokine/cytokine receptor genes after necropsy**.

Table 3 gives the number of larvae used for the investigation of the parasite morphogenesis. With the exception of IL-4R^-/- ^KO mice that kept the infection only for a few days (data not included in statistics below), larvae increased significantly (p < 0.001) in length and width with time (Figure 2 and 3). The greatest larval growth was observed in IL-4R^-/-^/IL-5^-/- ^DKO followed by IL-5^-/- ^KO, IFN-γ^-/-^/IL-5^-/- ^DKO and lastly IFN-γ^-/- ^KO. It was further observed that 30 days post infection; the larvae experienced a faster growth with a 2 to 3 fold increase in the length and width. Larvae recovered at day 10 were L4. All larvae recovered from day 30 onward were immature adults. Worms recovered 70 days post infection were still immature adults.

**Table 3:** Number of worms examined for measurement per strain of BALB/c mice at different necropsy time.

Mice Strains	IFN-y KO		IFN-y/IL-5 DKO		IL-5 KO		IL-4R/IL-5 DKO	
	**No. of mice dissected**	**No. of larvae used for measurement**	**No. of mice dissected**	**No. of larvae used for measurement**	**No. of mice dissected**	**No. of worms used for measurement**	**No. of mice dissected**	**No. of worms used for measurement**

**D2**	5	34	3	39	5	31	1	48
**D5**	5	21	3	13	7	15	3	85
**D10**	4	30	3	24	5	28	3	40
**D15**	3	3	4	3	6	15	4	88
**D20**	4	2	3	8	5	13	4	26
**D30**	3	-	3	4	4	3	3	6
**D40**	3	-	2	3	5	3	3	26
**D50**	4	-	2	-	4	5	2	14
**D60**	3	-		-	3	-	2	9
**D70**	-	-		-	4	-	2	2

	**34**	**90**	**23**	**94**	**48**	**113**	**27**	**344**

## Discussion

The fact that small laboratory animals are not permissive to *L. loa *motivated us to use knockout mice to study the survival and development of *L. loa *in the mammal host. The present results show a differential survival and development of *L. loa *in different strains of knockout mice. The strain that best accommodated *L. loa *was the IL-4R^-/-^/IL-5^-/- ^KO with larvae surviving up to 70 days post infection. The other strains kept the infection for 50 days (IL-5^-/- ^KO), 40 days (IFN-γ^-/-^/IL-5^-/- ^DKO), 20 days (IFN-γ^-/- ^KO) and 5 days (IL-4R^-/- ^KO). This differential survival and development of *L. loa *in cytokine knockout BALB/c mice emphasizes the importance of these classical Th1 and Th2 cytokines in the development and establishment of filarial worms in their vertebrate hosts. Even though wild type laboratory mice do not support the life cycle of human filariae, this study shows the importance of genetically modified mice (knockout mice) as experimental models to study the immune effectors regulating the survival and establishment of *L. loa *in a mammalian host.

In all strains of BALB/c mice used in this study, we observed a massive clearance of larvae a few days after infection. More than 50% of them were killed within the first 2 days of infection. The rapid clearance of filarial larvae inoculated into the mammalian host was first reported in *Monanema martini *infection in the striped grass mouse *Lemniscomys striatus *and also for *Litomosoides sigmodontis *in BALB/c mice and the jird, *Meriones unguiculatus *[21-24]. This may be attributed to the innate immune responses at the site of inoculation. It was estimated that about one third of the larvae that enter the lymphatic system, which is more permissive than blood or the muscular interstitial tissues, survive this non-specific immune clearance of larvae [21-24]. In the present study, the recovery of larvae in the muscles and internal organs (contained in the pleural and peritoneal cavities) 2 days post-inoculation are in accordance with the findings in the *Monanema *and *Litomosoides *experimental models.

Migrating larvae were found in the genital organs, peritoneal and pleural cavities, lungs and heart. This migration and localization of *L. loa *larvae is similar to what was observed in other studies with *L. loa *using immunodepressed BALB/c and Swiss mice, and with *L. sigmodontis *and *Brugia malayi *in Cotton rat and jird hosts respectively [16,25].

The measurement of the length and width of larvae recovered at various time points post inoculation showed a significant increase with time, indicating that the larvae effectively grew and developed. Larval growth was accelerated 20 to 30 days post infection. Larvae recovered after 30 days showed a 2 to 3-fold increase in length and width. The worms recovered 70 days post infection were immature adults and were not mature enough to produce microfilariae. *L. loa *reaches patency in the non-human primate 4 to 7 months post inoculation [26]. The data obtained in this study show that the development of *L. loa *is achievable in some knockout mice, however, it would be necessary to keep the worms for longer periods in these mice to observe further morphological changes, possible maturation and patency.

In all strains of mice, a large number of larvae (about 80%) remained in the subcutaneous tissue and the muscles, which are the sites of predilection of *L. loa *worms in the mammalian host. As was observed in previous studies [16,25], only a few larvae migrated to internal organs with no organ specific location. Larvae recovered from internal organs were found in the peritoneal and pleural cavities, heart and lungs. The presence of *L. loa *in the heart and lungs indicates that they may have used the thoracic duct to migrate to those organs as was demonstrated in *M. martini *in its natural host *L. striatus, L. sigmodontis *and *B. malayi *in their surrogate host *Meriones unguiculatus *[26,21,24]. If such cardio-pulmonary location of *L. loa *could occur in the human host, this might be associated with clinical manifestations such as the tropical pulmonary eosinophilia that is not presently known to be related to loiasis.

In this study, we observed that the double absence of IL-4R and IL-5 promoted the survival of *L. loa*. When IL-5 was singly knocked out, larvae survived up to 50 days. But when IL-5 and IL-4R were both knocked out, the larvae survived up to 70 days, suggesting an additive effect of IL-4R on IL-5 in the clearance of *L. loa *worms. These two cytokines have been previously associated with resistance of the host to infection. IL-4 and IL-5 in addition to IL-13 are required to prevent the development of adult worms as well as development of microfilaremia [17,27]. When IFN-γ in addition to IL-5 was knocked out, larvae survived only for 40 days. Double knockout of IFN-γ and IL-5 genes does not present an advantage for the development of *L. loa *compared to singly knocked out IL-5. This study clearly demonstrates that IL-5 is an important effector in the clearance of *L. loa *worms, the other cytokines (IFN-γ and IL-4) play a secondary role. IL-5 alone has been shown to control adult worm development in primary infection. In murine infection with *L. sigmodontis*, its deficiency leads to an increased parasite burden [17,28,29]. In other murine models, IL-5 played an important role during expression of protective immunity to *Onchocerca lienalis *infective larvae in mice [30]. In those studies, IL-5 was the dominant cytokine response coinciding with parasite clearance. In the *L. sigmodontis *model, it was also shown that the depletion of IL-5 leads to higher worm recovery and a higher level of microfilaraemia [29]. IL-5 appears to perform its function by recruiting eosinophils, which degranulate to kill the parasite [31].

## Conclusion

The data obtained shows that *L. loa *exhibits a differential survival and development in different strains of cytokine/cytokine receptor knockout BALB/c mice with the IL4R/IL-5 DKO accommodating the worms for longer period and provide better conditions for their growth. Findings of this study using the knockout mice approach have confirmed previous findings on the role of some cytokines in preventing this filarial infection in mice. This therefore opens an avenue for the exploitation of this approach to accelerate investigations to better understand the immune effectors important for the survival and development of *L. loa *in the mammalian host.

## List of abbreviations

IMMIP: Institute of Medical Microbiology, Immunology and Parasitology; SAE: Severe adverse events; KO: Knockout; RPMI: Roswell Park Memorial Institute medium1640; ANOVA: Analysis of variance; DKO: Double knockout; EFINTD: European Foundation Initiative for Neglected Tropical Diseases; EPIAF: Enhanced Protective Immunity against Filariasis

## Competing interests

The authors declare that they have no competing interests.

## Authors' contributions

NT participated in the study design, carried out laboratory experiments, analyzed the data and drafted the manuscript. SW participated in the study design, interpreted the results and edited the manuscript. JCN participated in production of infective larvae and dissection of mice. MEE participated in the production of infective larvae and dissection of mice. SS participated in the interpretation of the results and edited the manuscript. PE participated in the study design, and edited the manuscript. KIM: interpreted and edited the manuscript. AH participated in the study design, interpreted the results and edited the manuscript. All authors read and approved the final version of the manuscript.
